# Comparison of Catalyzing Properties of Bacterial 4-α-Glucanotransferases Focusing on Their Cyclizing Activity

**DOI:** 10.4014/jmb.2009.09016

**Published:** 2020-10-08

**Authors:** Jung-Eun Kim, Phuong Lan Tran, Jae-Min Ko, Sa-Rang Kim, Jae-Han Kim, Jong-Tae Park

**Affiliations:** 1 Department of Food Science and Technology, Chungnam National University, Daejeon 34734, Republic of Korea; 2 Department of Food Technology, An Giang University, An Giang, Vietnam; 3 Vietnam National University, Ho Chi Minh, Vietnam; 4 Department of Food Nutrition, Chungnam National University, Daejeon 373, Republic of Korea

**Keywords:** 4-α-Glucanotransferase, cycloamylose, cyclization, disproportionation, cyclic maltopentaose

## Abstract

A newly cloned 4-α-glucanotransferase (αGT) from *Deinococcus geothermalis* and two typical bacterial αGTs from *Thermus scotoductus* and *Escherichia coli* (MalQ) were investigated. Among 4 types of catalysis, the cyclization activity of αGTs that produces cycloamylose (CA), a valuable carbohydrate making inclusion complexes, was intensively studied. The new αGT, DgαGT, showed close protein sequence to the αGT from *T. scotoductus* (TsαGT). MalQ was clearly separated from the other two αGTs in the phylogenetic and the conserved regions analyses. The reaction velocities of disproportionation, cyclization, coupling, and hydrolysis of three αGTs were determined. Intriguingly, MalQ exhibited more than 100-fold lower cyclization activity than the others. To lesser extent, the disproportionation activity of MalQ was relatively low. DgαGT and TsαGT showed similar kinetics results, but TsαGT had nearly 10-fold lower hydrolysis activity than DgαGT. Due to the very low cyclizing activity of MalQ, DgαGT and TsαGT were selected for further analyses. When amylose was treated with DgαGT or TsαGT, CA with a broad DP range was generated immediately. The DP distribution of CA had a bimodal shape (DP 7 and 27 as peaks) for the both enzymes, but larger DPs of CA quickly decreased in the DgαGT. Cyclomaltopentaose, a rare cyclic sugar, was produced at early reaction stage and accumulated as the reactions went on in the both enzymes, but the increase was more profound in the TsαGT. Taken together, we clearly demonstrated the catalytic differences between αGT groups from thermophilic and pathogenic bacteria that and showed that αGTs play different roles depending on their lifestyle.

## Introduction

4-α-Glucanotransferase (αGT, E.C. 2.4.1.25) has intermolecular and intramolecular transglycosylation activities and is involved in cleaving α-1,4-glycosidic linkages and generating new α-1,4-linkages. This type of enzyme is widely distributed in bacteria, and similar enzymes are reported from some plants such as *Solanum tubersum* [1-4]. Thermostable αGTs from *Thermus* species were intensively studied for decades, and some *Thermus* αGTs were successfully applied to modifications of starch or starch derivatives [5-8]. For example, thermo-reversible starch gel was made of clusters that were generated by αGT treatment on amylopectin [5]. On the other hand, MalQ, an αGT-type enzyme in many pathogenic bacteria, has been investigated to elucidate its role in the carbohydrate metabolism and pathogenesis of bacteria [9-11]. *Esherichia coli* MalQ has crucial roles in maltose/maltodextrin metabolism. MalQ is the only enzyme that utilizes maltose in *E. coli* [12]. Recently, we clearly demonstrated its role in the degradation of glycogen [1].

Catalytic reactions that are conducted by αGTs are classified into 4 types, disproportionation, cyclization, coupling, and hydrolysis [13]. Usually, the three transglycosylation activities are much stronger than the hydrolysis in common bacterial αGTs. When αGT cleaves an α-1,4-glycosidic bond and transfers the donor glycan in the α-1,4-bond to the non-reducing end of another glucan, that is the disproportionation reaction. The coupling reaction is different from the disproportionation, because it uses cyclic glucans as a donor molecule. Cyclization is an intramolecular transglycosylation. Well-known commercial products produced by this cyclization activity are cyclodextrins (CDs) [3, 13].

Cycloamylose (CA) is a cyclic oligosaccharide composed of α-1,4-glycosidic linkages, exclusively. Low degrees of polymerization (DPs) ranging from DP 6 ~ 8 result in α-, β-, and γ-CD, respectively. Generally, the terminology regarding CA includes broad DP ranges from small CDs to large molecules having DPs above 50. CDs have a hydrophobic central cavity and accommodate various guest molecules that form an inclusion complex and change their solubility, reactivity, or stability. CA, which contains cyclic glucans with inner cavities of various size, is also used as a host molecule for inclusion complex and protein refolding agent [14, 15].

Many αGTs have been reported but no research on the kinetic analyses of four different types of catalysis (disproportionation, cyclization, coupling, and hydrolysis) has been published. Therefore, in this study, we determined the reaction velocities of four types of catalysis using three bacterial αGTs including a newly cloned αGT from *Deinococcus geothermalis* and well-characterized αGTs from *T. scotoductus* and *E. coli*. Assay methods for precise, direct detection of each catalysis type were successfully developed and applied. In addition, our intensive analyses on the cyclization reaction and CA production will provide valuable information for further practical research using αGTs.

## Materials and Methods

### Protein Sequence Analysis

Protein sequence analysis was carried out using the BLAST sequence analysis tool as described by Altschul *et al*.[16] with the gi number of each microorganism as follows: gi|38505481|_*Thermus aquaticus*, gi|570754797|_*Thermus thermophilus*, gi|320150052|_*T. scotoductus*, gi|310689644|_*Thermus brockianus*, gi|94555055|_*D. geothermalis*, gi|653292780|_*Deinococcus radiodurans*, gi|490649772|_*Geobacter metallireducens*, gi|18159937|_*Pyrobaculum aerophilum*, gi|446993535|_*Vibrio cholerae*, gi|647458543|_*Vibrio parahaemolyticus*, gi|695739988|_*Enterobacter cloacae*, gi|740849651|_*Citrobacter freundii*, gi|82778768|_*Shigella dysenteriae*, gi|16131292|_*E. coli*, gi|110616799|_*Shigella flexneri*.

### Expression and Purification of αGT Enzymes

*Escherichia coli* MC1061 and BL21, the transformant hosts carrying the recombinant plasmids, were cultured overnight in LB medium containing ampicillin (100 μg/ml) at 37°C with shaking. After harvesting, the cell pellets were resuspended in lysis buffer [50 mM Tris-HCl (pH 7.5), 300 mM NaCl, 10 mM imidazole] and then sonicated in an ice bath using sonicator (sonicator ultrasonic processor, Osonica, USA, 2.5 min × 4 times). The crude cell extracts were purified by FPLC Ni-NTA affinity chromatography with Ni-NTA resin (Qiagen, USA) packed in a column (Bio-Rad, USA). The purity and activity of the enzymes were determined by SDS-PAGE and Lugol’s method, respectively [8, 17].

### Determination of Velocity of Four Types of αGT Reaction Types

The disproportionation and coupling activities of the αGTs were assayed by the decrease of glucose amount using the glucose oxidase-peroxidase (GOPOD) method [18]. The reaction mixtures were prepared with 0.5% (w/v) amylose and 0.025% (w/v) glucose for disproportionation and 0.5% (w/v) cycloamylose and 0.02% (w/v) glucose for coupling in 50 mM sodium acetate buffer (optimum pH 7.5 and 6.5 for TsαGT and both DgαGT and MalQ, respectively). They were separately incubated with 100 μl of each enzyme at optimum temperature of 37°C, 55°C and 75°C for MalQ, DgαGT and TsαGT, respectively. After cooling down, a 100 μl aliquot of each reaction was mixed with 1 ml of GOPOD solution, incubated at 37°C for 30 min and then the absorbance was measured at 510 nm. The disproportionation or coupling activities of enzymes were defined as the amount of enzyme that can decrease 1 μmole of glucose per min.

The cyclization activity of the αGTs was measured by the increase of cycloamylose product using high-performance anion exchange chromatography (HPAEC) [19]. The reaction mixtures containing 0.5% (w/v) amylose in 50 mM sodium acetate buffer using optimum pH as described above were severally incubated with 100 μl of each enzyme at optimum temperature as also described above. After enzyme reaction, the 100 μl aliquots of the reaction mixtures were then treated with β-amylase to degrade linear maltooligosaccharides into maltose. The β-amylase-resistant cyclic glucans were isolated by ethanol precipitation and analyzed by HPAEC. The cyclization activity of enzymes was defined as the rate at which a certain enzyme amount can synthesize cycloamylose per min.

The hydrolysis activity of the αGTs was determined by the increase of reducing sugar using the copper-bicinchoninate method [20]. The reaction mixtures containing 0.5% (w/v) amylose in 50 mM sodium acetate buffer at optimum pH were incubated with 100 μl of each enzyme at optimum temperature. The enzyme reactions were eliminated by a same sample volume of 0.1 N NaOH. Then, 200 μl aliquot of each enzyme reaction mixture was added with an equal amount of copper-bicinchoninate reagent and incubated at 80°C for 35 min and at which a certain enzyme amount at 575 nm. The hydrolysis activity of enzymes was defined as the rate at which a certain enzyme amount can hydrolyze the substrate into reducing end sugar per min.

### Production of CA

To produce CA, 0.1% (w/v) amylose was separately incubated with 0.05 U/mg of purified DgαGT and TsαGT in 50 mM sodium acetate buffer at optimum temperature. The reaction mixtures were treated with β-amylase to remove small maltodextrins. The CA products were then isolated by ethanol precipitation and centrifugation. The CA pellets were dried and resuspended in distilled water for further analysis.

### High-Performance Anion-Exchange Chromatography (HPAEC) Analysis

The side chain distribution of modified starch was determined by HPAEC. The enzyme reaction mixtures were stopped by boiling for 5 min and centrifuged at 13,000 ×g and 4°C for 1 min. The supernatants were filtered through 0.45 μm membranes and then injected into the HPAEC system (DX-500 system, Dionex, USA) with a pulsed electrochemical detector (ED40, Dionex). The ingredients of samples were separated in a CarboPac PA1 column (250 × 4 mm, Dionex) at a flow rate of 1 ml/min with a 10-64% (v/v) gradient of 600 mM sodium acetate in 150 mM NaOH.

### Matrix-Assisted Laser Desorption/Ionization Time-of-Flight/Mass Spectroscopy (MALDI-TOF/MS) Analysis

MALDI-TOF/MS analysis of cycloamylose products was carried out in positive ion reflectron mode using an UltrafleXtreme system (Bruker Daltonics, USA). One microliter of cycloamylose solution was spotted onto a stainless steel target plate and followed by 0.3 μl of 0.01 M NaCl and 0.5 μl of 5% 2,5-dihydroxybenzoic acid in 50%acetonitrile. The spot was rapidly dried under vacuum for more homogenous crystallization. Mass spectra were externally calibrated using maltooligosaccharides isolated from commercial beer. Raw MS data was processed with FlexAnalysis software (version 3.3, Bruker Daltonics) [21].

## Results and Discussion

### Sequence Analysis of Three αGTs

**Fig. 1:**
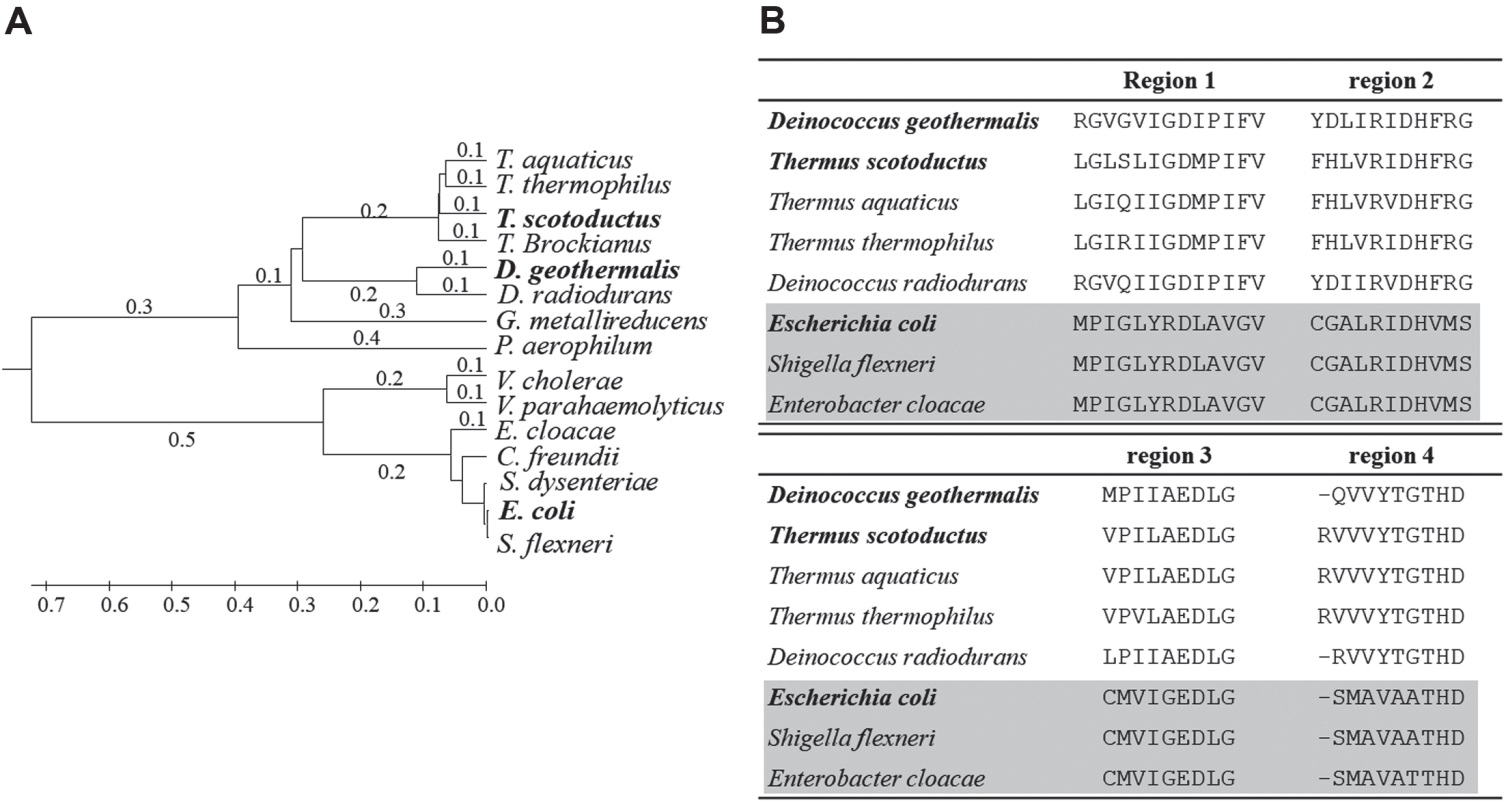
Phylogenic tree of 4αGT protein sequences (A). The tree was built by the neighbor-joining method and implemented in the CLUSTALW program. Comparison of amino acid sequences in the 4 conserved regions of various 4αGTs (B).

Protein sequences of DgαGT, TsαGT, and MalQ were compared by conducting phylogenetic tree analysis with other αGTs from thermophilic or pathogenic bacteria (Fig. 1A). DgαGT showed the primary structure of the enzyme similar to the cluster of αGTs from genus *Thermus*. In contrast, MalQ was completely separated from the other two enzymes and clustered with αGTs from *Shigella* species. Intriguingly, the sequences from *E. coli* and *S. flexneri* αGTs are nearly identical (99.4%, determined by BLAST provided by www.blast.ncbi.nlm.nih.gov). The phylogenetic tree was roughly divided into two big clusters of proteins: one group from thermophilic bacteria and the other from pathogenic bacteria. MalQ exhibits very high sequence homology with other αGTs from pathogenic bacteria, such as *S. dysenteriae*, *C. freundii*, and *En. cloacae*. In analysis of four well-conserved regions, αGTs from *E. coli*, *S. flexneri*, and *En. cloacae* have exactly the same sequences at all four regions (Fig. 1B). Among genus *Thermus*, the αGT sequences are strongly conserved by comparison with other carbohydrate enzymes such as glycogen branching enzymes [22].

It is plausible that horizontal αGT gene transfer happened among the pathogenic bacteria. In addition, the αGT sequences from *Thermus* were no different from their common ancestor. The conservation of the protein sequences strongly indicates a crucial role of αGTs in those bacteria. The role of αGT in the thermophilic bacteria is probably different from that for the pathogens due to the profound differences in their lifestyles. In the next section, this postulation was proved by intensive analyses of the 4 types of catalysis conducted by the 3 αGTs.

### Determination Kinetic Parameters of Four Types of Catalysis

**Fig. 2:**
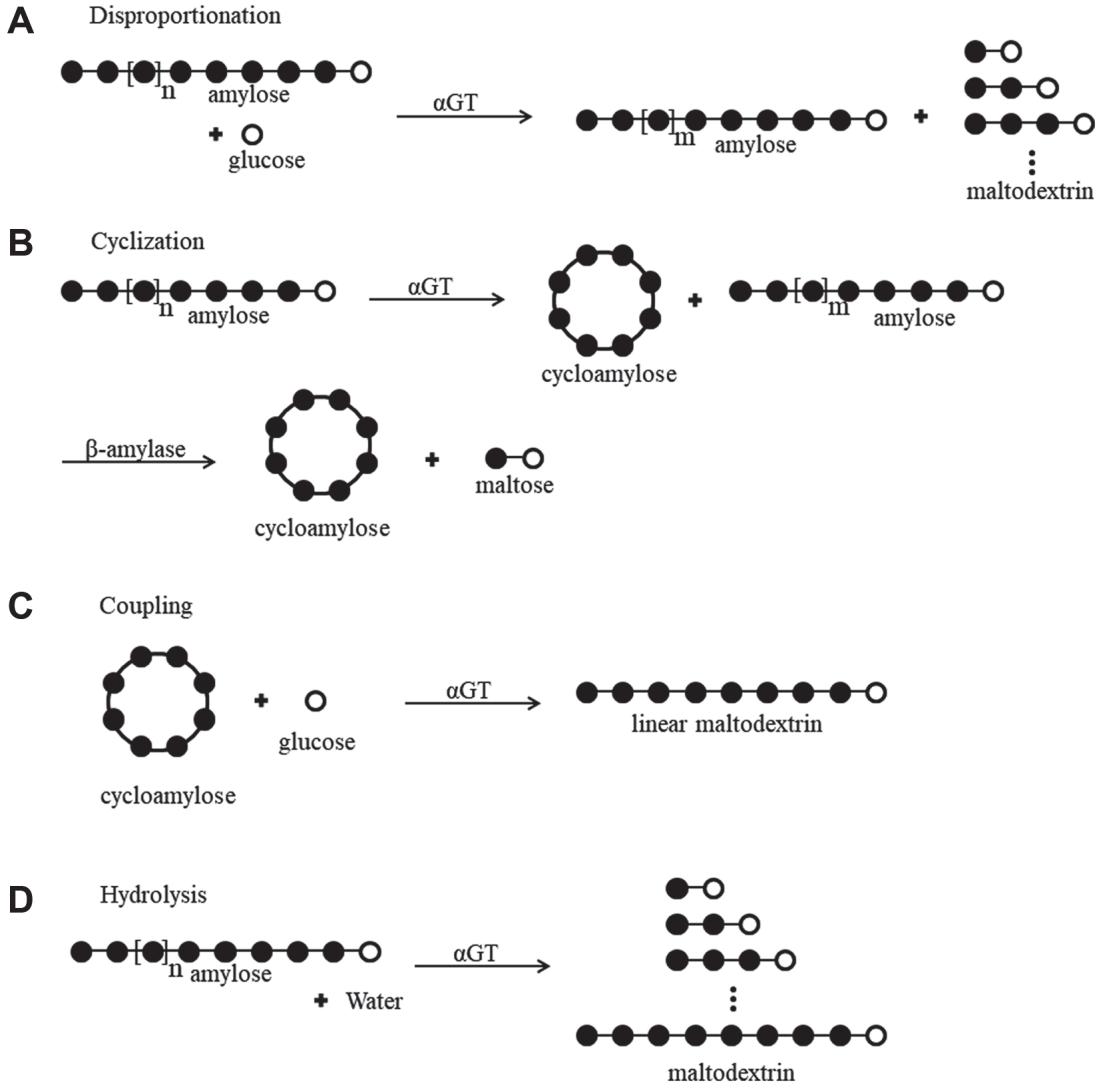
Schematics of the four reactions catalyzed by 4αGT. Schematics of the four reactions catalyzed by 4αGT. (**A**) Disproportionation: the enzyme cleaves and transfers the α-1,4-linkages of saccharides to acceptor molecule. Amylose is used as the donor molecule and glucose as the acceptor molecule. (**B**) Cyclization: the non-reducing end of saccharides is used as the acceptor resulting in circular saccharides with α-1,4-linkages, cycloamyloses. (**C**) Coupling: the cycloamylose ring is cleaved and transferred to a glucose, the acceptor molecule, then linear product is generated. The cycloamylose is used as the donor, and the glucose as the acceptor. (**D**) Hydrolysis: a water molecule replaces the saccharide and shorter saccharides are produced. The black and white circles indicate the anhydroglucose residues and the reducing end glucoses, respectively.

The disproportionation, cyclization, coupling, and hydrolysis activities of the αGTs were determined using more quantitative methods than conventional ones. The assays were designed to quantitatively measure the number of catalytic reactions by determining increase of products or decrease of reactants. Basically, saturation kinetics were conducted to determine reaction velocities. The reaction mechanisms of the four types of catalysis and scheme of the detection methods are shown in Fig. 2. The detailed conditions are given in the Materials and Methods section.

**Fig. 3:**
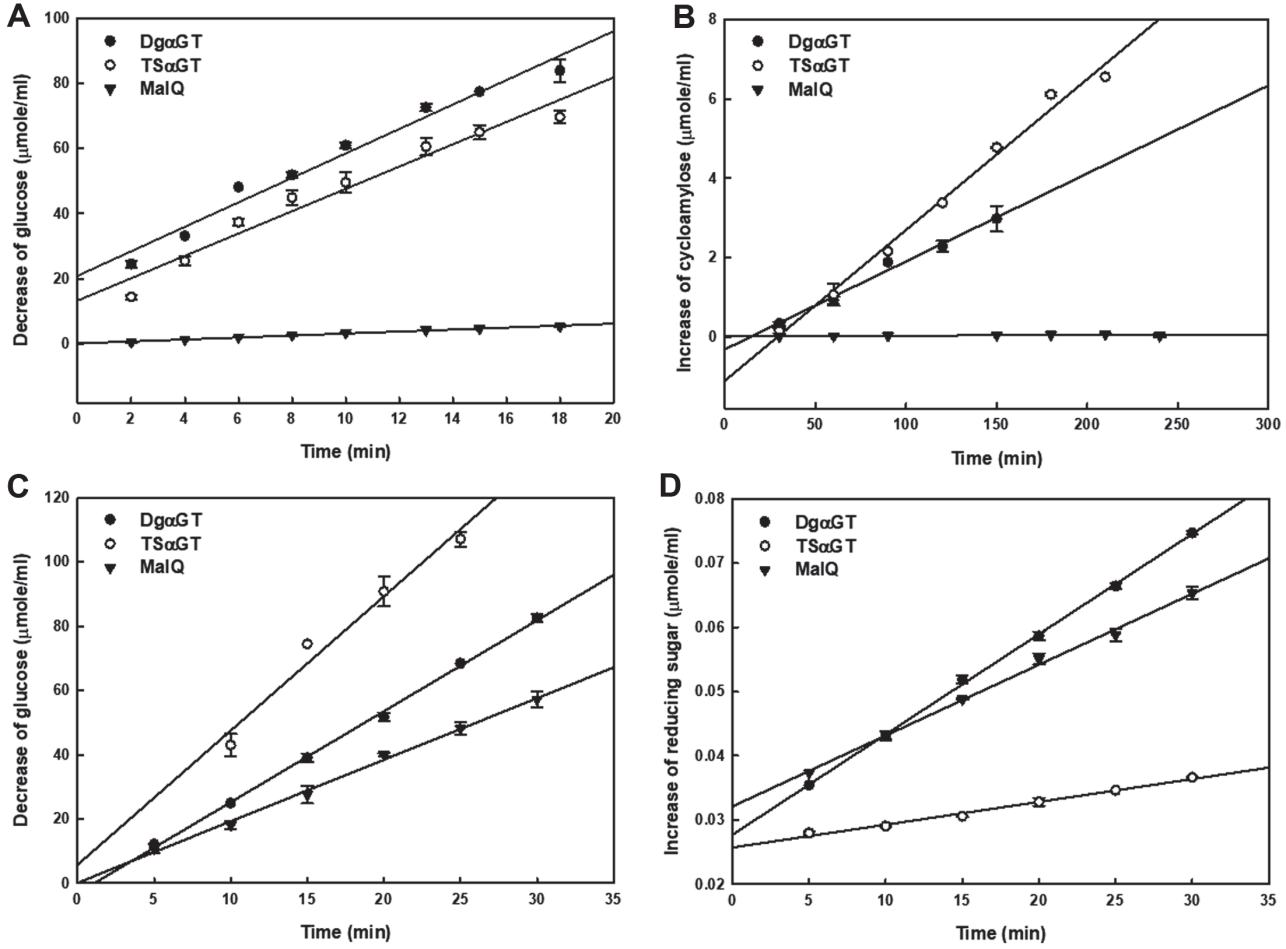
Determination of reaction velocity for the disproportionation (A), the cyclization (B), the coupling (C), and the hydrolysis (D). Note that x-y axis of all graphs has the same unit.

Overall, three enzymes had strong activity on intermolecular tranglycosylation (disproportionation, coupling) compared with intramolecular reaction (cyclization) (Fig. 3, Table 1). Also, the hydrolysis activity was at least 1,000-fold lower than the tranglycosylation activities in all the enzymes. DgαGT and TsαGT exhibited similar catalytic properties, but TsαGT had about 5-fold greater transglycosylation/hydrolysis ratio. MalQ, which was completely separated from the others in the phylogenetic tree, showed extremely low activity for the cyclization. The coupling and the hydrolysis activities of MalQ were comparable to those of DgαGT, but the enzyme had disproportionation activity below one twelfth that of DgαGT. The results clearly indicate that αGTs from thermophilic bacteria, DgαGT and TsαGT, differently contribute to the maltose/maltodextrin metabolism from MalQ in *E. coli* [12].

When the activities are expressed relatively to the cyclization, the differences between the two αGT groups are more prominent. In the *E. coli*-type lifecycle, cycloamylose probably has no meaningful role. Even though β-CD was detected from *E. coli* cell lysate (data not shown), the concentration was very low compared with other linear maltodextrins. The αGTs from thermophilic bacteria accumulated a greater amount of CA, when amylose or maltodextrin with a long DP was used as a substrate [7]. It is plausible that the enzymes accumulate CAs, and the cyclic glucans are recycled depending on bacterial metabolic phase. The accumulation of CAs in the cytosol of thermophilic bacteria can help with folding of proteins [23, 24]. In a practical approach, TsαGT might be a very good enzyme to produce CA. The enzyme has very low hydrolysis activity. However, relatively high coupling activity can disturb the accumulation of CA in high concentration. To solve this problem, one could try to continuously remove small maltodextrins that serve as receptors in the coupling reaction.

### Cyclization Activity and Cycloamylose Analyses

**Fig. 4:**
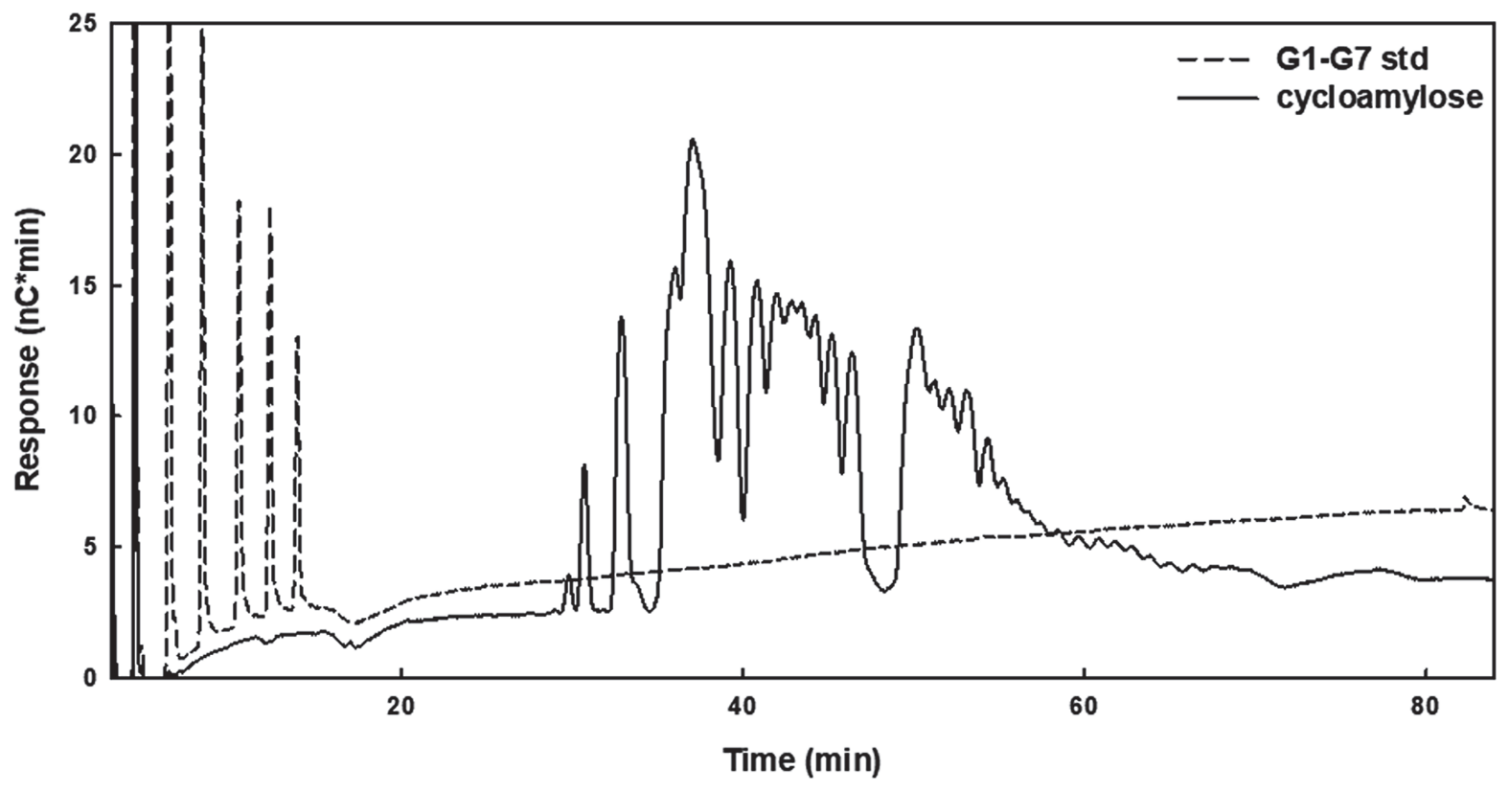
Cycloamylose analysis produced by DgαGT using amylose as a substrate. At maximum yield of cycloamylose production (6 h reaction), the reactant was treated by β-amylase to remove linear saccharides, then analyzed by HPAEC.

To further investigate the cyclization properties of the αGTs, long-term reactions using amylose as a substrate were conducted for DgαGT and TsαGT. MalQ had too low cyclization activity to be used. The cyclization reaction was continued to the equilibrium point where no more significant change of products was observed. DgαGT produced a significant amount of CA from amylose, and the maximum yield was about 45% at 6 h (Fig. 4). Intriguingly, TsαGT showed much slower production and reached maximum after 24 h. The yield of TsαGT was similar to that of DgαGT. Further optimization might be needed to increase the CA production yield by TsαGT.

**Fig. 5:**
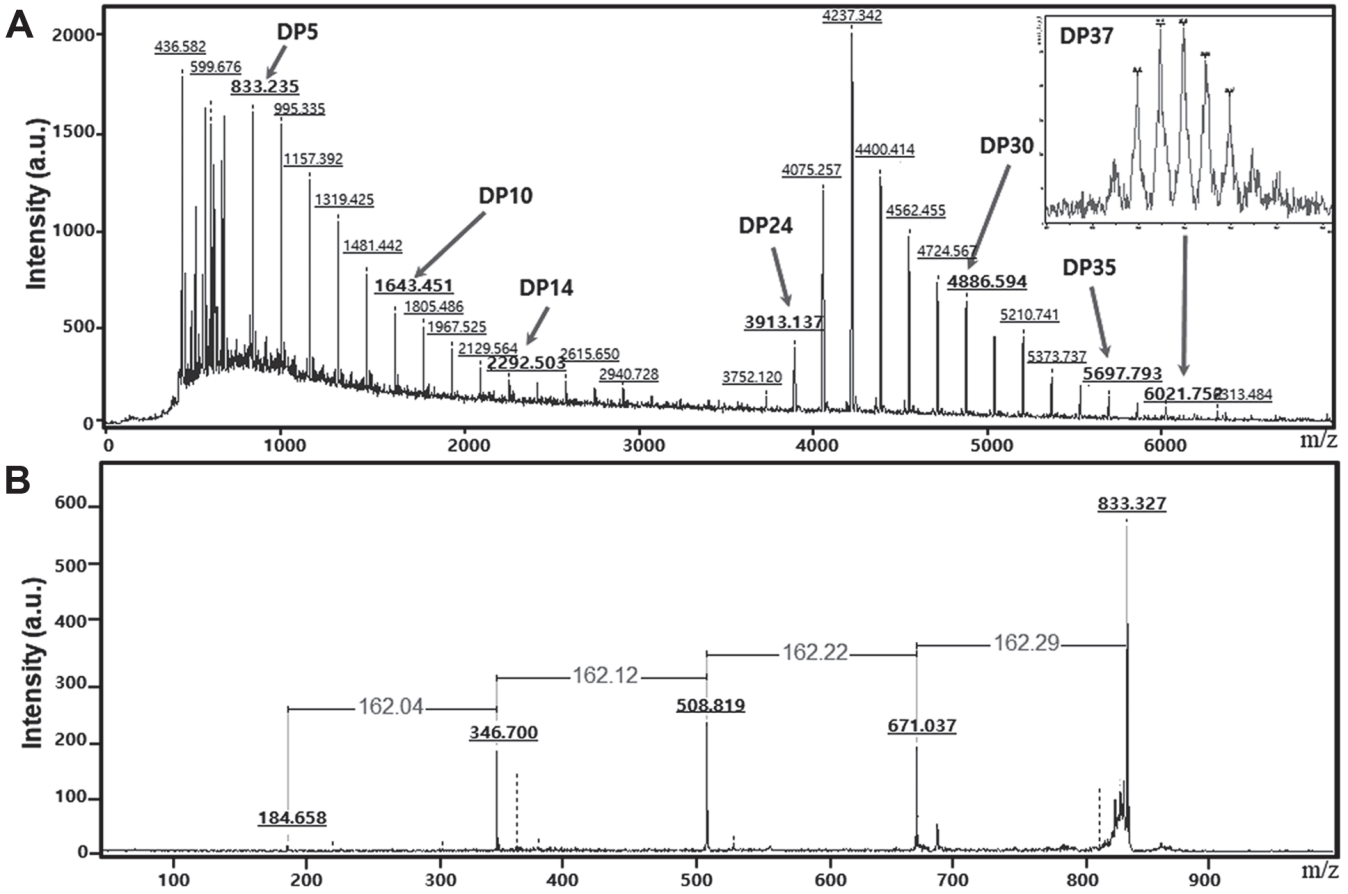
MALDI-TOF/MS analysis of cycloamyloses produced by DgαGT. The number on each peak indicates the molecular mass (**A**). CDP5 was generated profoundly. Tandem mass spectrometry analysis reveals that the peak with Mw 833.235 was CDP 5 product which consists of anhydroglucoses (**B**).

**Fig. 6:**
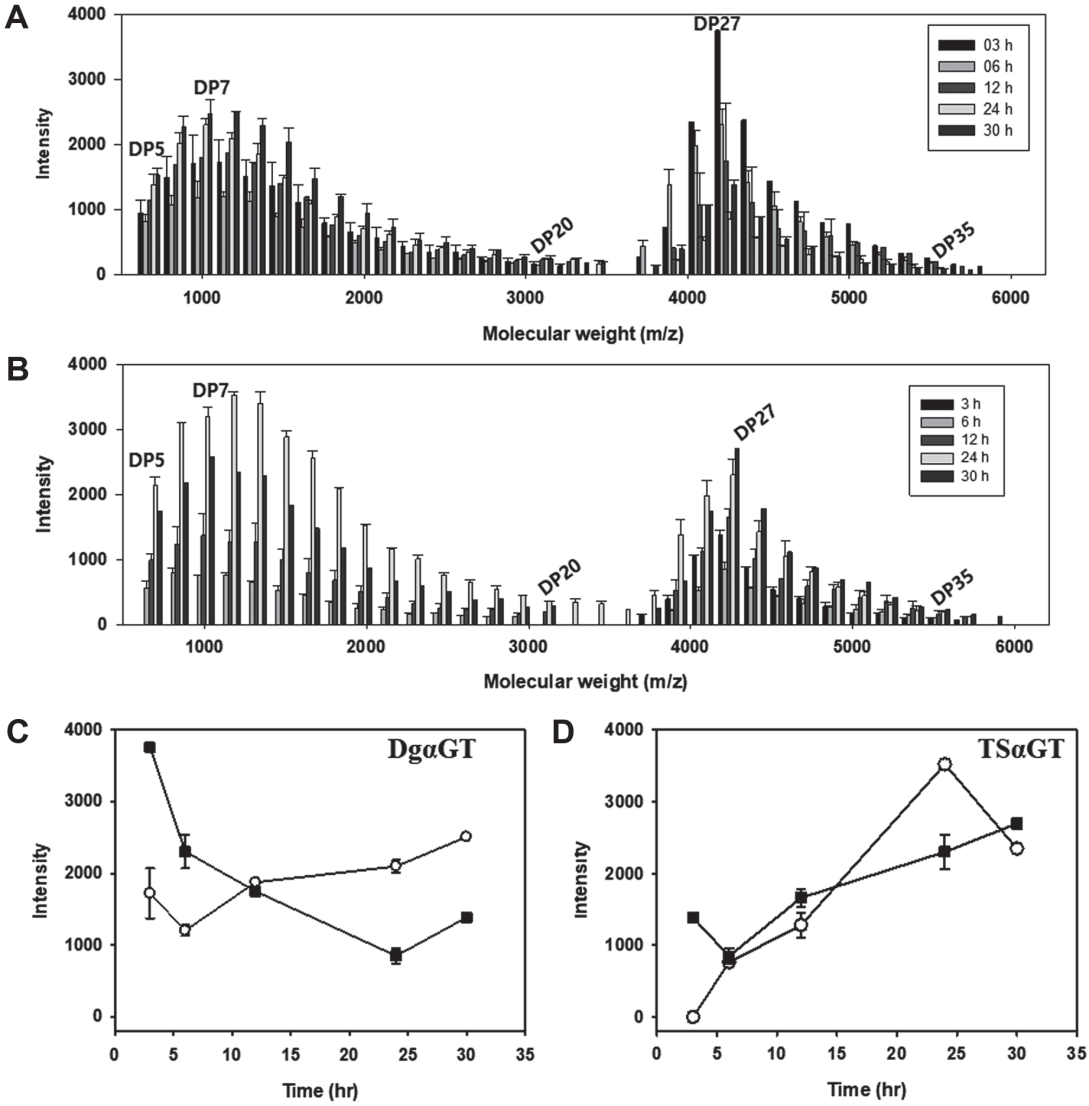
Composition of DP5-35 cycloamyloses produced by DgαGT (A) and TSαGT (B). Note, no cycloamylose under DP22 was detected at 3 h reaction sample of TSαGT. Quantitative variation of DP7 (white circle) and DP27 (black square) cycloamayloses during the reaction are compared at (C) and (D).

The CA distribution and peak shapes were similar in MALDI-TOF/MS chromatograms. The bimodal shapes appeared from early stage of CA synthesis for both enzymes. DP 6~ 8 and DP 27 were detected as peak DPs in the distribution (Fig. 5). The larger DP CA, especially CD 27, quickly decreased as the reaction continued in the CA product from DgαGT, and proportion of the smaller CA increased fast. In TsαGT’s case, the small CA increased faster than that of DgαGT but the amount of large CA also, to lesser extent, increased (Fig. 6). The large CA was used as substrates for the coupling reaction in the DgαGT reaction. TsαGT uses large CA for the coupling reaction, too. But the effect was not significant due to the ratio between the small CA and the larger CA at the early reaction stage. The CA distributions showed typical U-shapes with very low amount of the mid-size CA (DP 15~24) (Fig. 6). Steric hindrance by a protruding structure at the middle of the catalytic site of αGTs was suggested as the mechanism of this DP selectivity [25].

Cyclomaltopentaose (CDP5), a rare cyclic glucan, was detected at the early of reaction and accumulated through the reaction. The enzymes produced a large amount of CDP5 similar to the amount of CDs (Fig. 5B). In previous studies, CDP5 with production of all α-1,4-linkages was not successfully detected. A computer modeling study reported that the torsion angle in CDP5 was not possible in the enzyme synthesis [26]. However, our results clearly indicate CDP5 production is able to be achieved by DgαGT and TsαGT.

In brief, all three enzymes showed their higher activity in catalysis of disproportionation and coupling than cyclization and hydrolysis. MalQ, thoroughly different in phylogenetic analysis from thermophilic bacteria but profoundly homologous to pathogenic bacteria, had a fairly low cyclization activity in comparison with DgαGT and TsαGT. CA products with bimodal shape of DP distribution were produced similarly by DgαGT and TsαGT, but not really much by MalQ. It is notable that TsαGT did not produce short DP CA at the early reaction stage. Among these CAs, cyclomaltopentaose was formed at early reaction stage and accumulated over the course of time, especially in TsαGT catalysis. This study should provide valuable insight for production of CA with food and pharmaceutical industry applications.

## Figures and Tables

**Table 1:** The reaction velocities for 4 types of catalysis were compared as unit per nmole of protein.

Reaction velocity (U/nmole protein)					

	Disproportionation	Cyclization	Coupling	Hydrolysis	Transglycosylation/Hydrolysis

DgαGT	3.75	2.21 × 10^-2^	2.83	1.56 × 10^-3^	4.23 × 10^3^
TSαGT	3.43	3.79 × 10^-2^	4.18	3.55 × 10^-4^	2.15 × 10^4^
MalQ	0.30	1.66 × 10^-4^	1.92	1.10 × 10^-3^	2.01 × 10^3^


Relative activity to cyclization					

	Disproportionation	Cyclization	Coupling	Hydrolysis	

DgαGT	1.70 × 10^2^	1	1.28 × 10^2^	7.05 × 10^-2^	
TSαGT	9.03 × 10	1	1.10 × 10^2^	9.36 × 10^-3^	
MalQ	1.82 × 10^3^	1	1.16 × 10^4^	6.67	

Relative values to cyclization activity are also given in a lower table. For the relative activities, comparison of the values from different enzymes are not proper.
